# (4*S*)-4-[(*R*)-Chloro­(4-nitro­phen­yl)meth­yl]-1,3-oxazolidin-2-one

**DOI:** 10.1107/S1600536813010398

**Published:** 2013-04-24

**Authors:** V. Gaumet, C. Denis, M. Madesclaire, V. P. Zaitsev

**Affiliations:** a UMR 990, INSERM, Université d’Auvergne, Laboratoire de Chimie Physique, Faculté de Pharmacie, 63001 Clermont-Ferrand, France; b Laboratoire de Chimie Thérapeutique, Faculté de Pharmacie, Université d’Auvergne, 63001 Clermont-Ferrand, France; c Samara State University, 433011 Samara, Russian Federation

## Abstract

In the title compound, C_10_H_9_ClN_2_O_4_, the oxazolidinone ring adopts a near-planar conformation, with mean and maximum deviations of 0.0204 (8) and 0.0328 (8) Å, respectively. The nitro group is twisted slightly from the plane of the benzene ring, making a dihedral angle of 6.79 (3)°. The dihedral angle between the mean oxazolidinone plane and the benzene ring is 56.21 (3)°. In the crystal, N—H⋯O hydrogen bonds and N—O⋯π inter­actions [O⋯centroid distances = 3.478 (1) and 3.238 (1) Å] dominate the packing, forming infinite zigzag chains along the *b*-axis direction. Neighbouring chains are linked together through C—H⋯O and C—H⋯Cl inter­actions. The absolute configuration of the two stereogenic centres was determined using the anomalous dispersion of the Cl atom.

## Related literature

For the biological activity of oxazolidinone derivatives, see: Michalska *et al.* (2012 ▶); Mathur *et al.* (2013 ▶); Jindal *et al.* (2013 ▶). For related structures, see: Bach *et al.* (2001 ▶); Tsui *et al.* (2013 ▶). For detailed of the synthesis, see: Madesclaire *et al.* (2013 ▶).
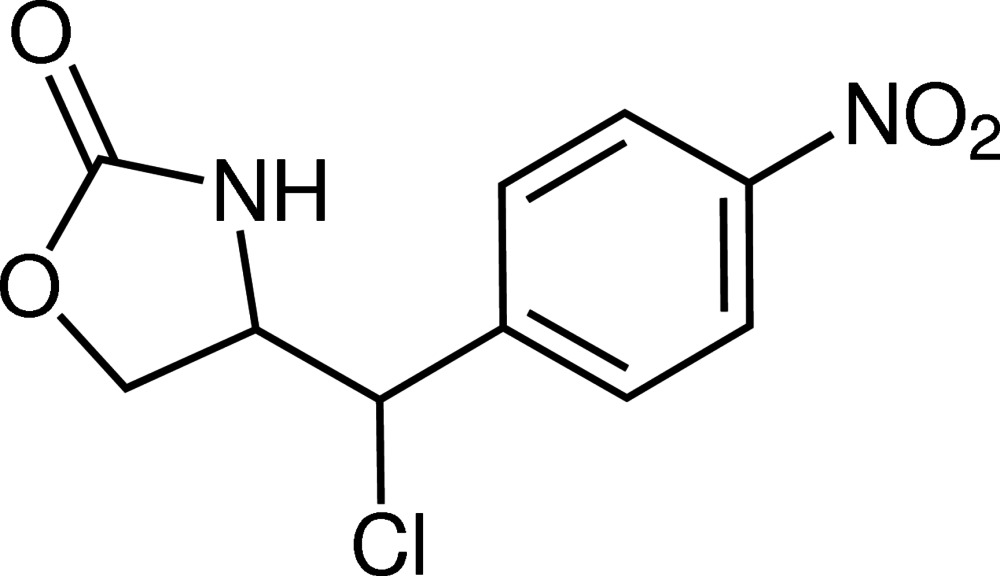


## Experimental

### 

#### Crystal data

C_10_H_9_ClN_2_O_4_
*M* *_r_* = 256.64
Monoclinic, 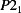

*a* = 7.2372 (1) Å
*b* = 6.6726 (1) Å
*c* = 11.7126 (2) Å
β = 106.715 (1)°
*V* = 541.71 (1) Å^3^
*Z* = 2
Mo *K*α radiation
μ = 0.36 mm^−1^
*T* = 296 K
0.52 × 0.49 × 0.34 mm

#### Data collection

Bruker APEXII CCD diffractometer
Absorption correction: multi-scan (*SADABS*; Bruker, 2012 ▶) *T*_min_ = 0.915, *T*_max_ = 1.000
12895 measured reflections
6114 independent reflections
5384 reflections with *I* > 2σ(*I*)
*R*_int_ = 0.014

#### Refinement

*R*[*F* ^2^ > 2σ(*F* ^2^)] = 0.037
*wR*(*F* ^2^) = 0.098
*S* = 1.06
6114 reflections
158 parameters
1 restraint
H atoms treated by a mixture of independent and constrained refinement
Δρ_max_ = 0.33 e Å^−3^
Δρ_min_ = −0.44 e Å^−3^
Absolute structure: Flack (1983 ▶), 2348 Friedel pairs
Flack parameter: −0.03 (3)

### 

Data collection: *APEX2* (Bruker, 2012 ▶); cell refinement: *SAINT* (Bruker, 2012 ▶); data reduction: *SAINT*; program(s) used to solve structure: *SHELXS97* (Sheldrick, 2008 ▶); program(s) used to refine structure: *SHELXL97* (Sheldrick, 2008 ▶); molecular graphics: *ORTEP-3 for Windows* (Farrugia, 2012 ▶) and *PLATON* (Spek, 2009 ▶); software used to prepare material for publication: *publCIF* (Westrip, 2010 ▶).

## Supplementary Material

Crystal structure: contains datablock(s) global, I. DOI: 10.1107/S1600536813010398/kp2451sup1.cif

Structure factors: contains datablock(s) I. DOI: 10.1107/S1600536813010398/kp2451Isup2.hkl

Supplementary material file. DOI: 10.1107/S1600536813010398/kp2451Isup3.cml

Additional supplementary materials:  crystallographic information; 3D view; checkCIF report

## Figures and Tables

**Table 1:** Hydrogen-bond geometry (Å, °)

*D*—H⋯*A*	*D*—H	H⋯*A*	*D*⋯*A*	*D*—H⋯*A*
N3—H3⋯O15^i^	0.77 (2)	2.32 (2)	3.095 (1)	179 (2)
C6—H6⋯O16^ii^	0.98	2.46	3.309 (2)	145
C11—H11⋯Cl17^iii^	0.93	2.83	3.582 (1)	139

Symmetry codes: (i) 
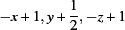
; (ii) 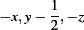; (iii) 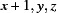.

**Fig. 1.**
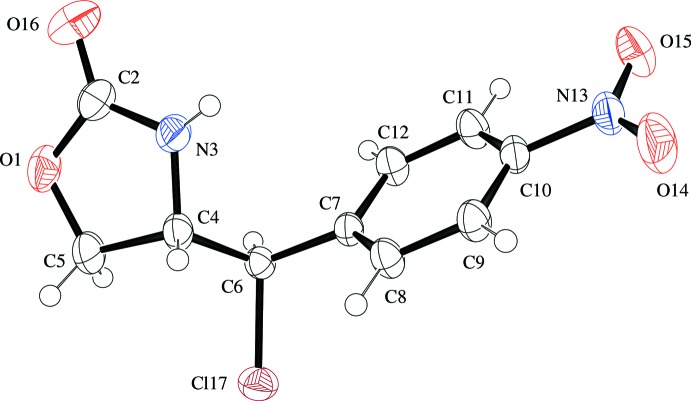
Molecular structure showing the atom labelling scheme and 50% probability displacement ellipsoids for non-H atoms.

**Fig. 2.**
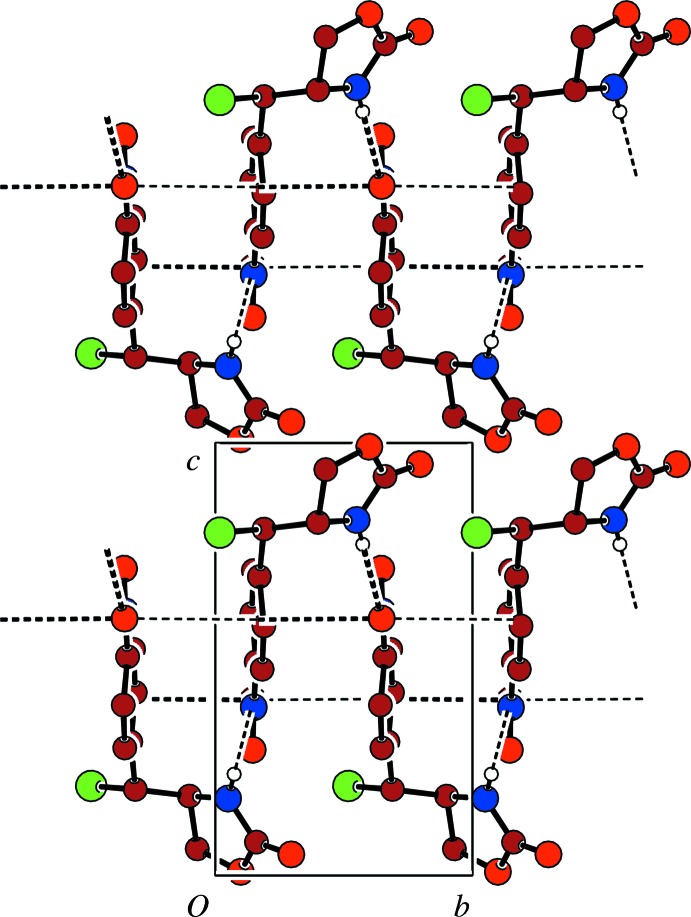
Projection along a axis, showing zigzag chain of C10H9ClN2O4 molecules connected by N—H···O hydrogen bonds and N—O···π interactions (dashed lines). H atoms not involved in hydrogen bonding have been omitted for clarity.

**Fig. 3.**
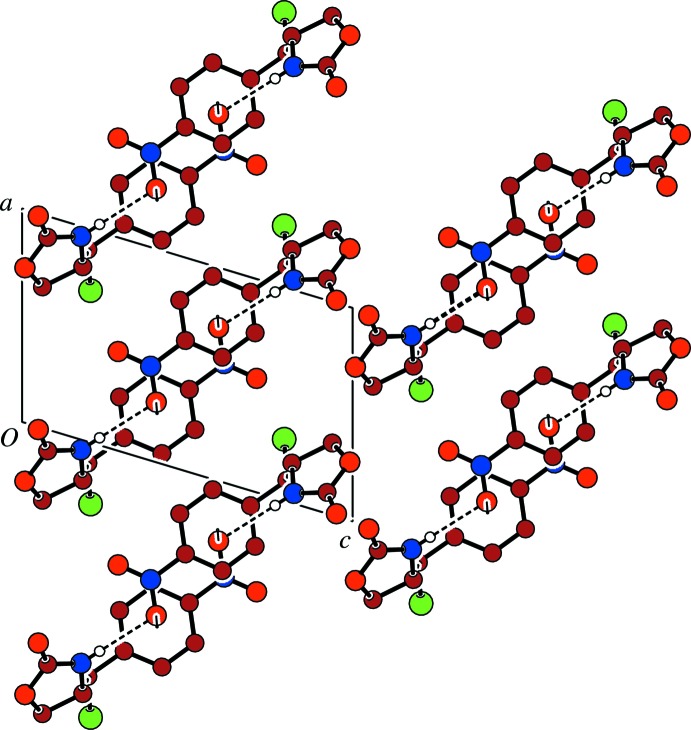
Projection along b axis, showing shift between adjacent chains. N—H···O hydrogen bonds and N—O···π interactions are represented as dashed lines. H atoms not involved in hydrogen bonding have been omitted for clarity.

## supplementary crystallographic information

### Comment

Oxazolidinones are a new class of synthetic antimicrobial agents. Linezolid is the first oxazolidinone approved for human use in the treatment of multidrug-resistant gram-positive bacterial infections (Michalska *et al.*, 2012; Mathur *et al.*, 2013). Linezolid may also offer novel disease modifying and symptomatic therapeutic potential for the treatment of anxiety disorders (Jindal *et al.*, 2013).

The molecular structure of the title compound (Fig. 1) reveals a planar oxazolidinone ring with a mean deviation of 0.0204 (8) Å, maximum deviation from planarity being 0.0328 (8) Å for atom C5. The dihedral angle between the mean oxazolidinone plane and the phenyl ring is 56.21 (3)°. The *p*-NO_2_ group form a interplanar angle of 6.79° with the benzene ring. In the crystal, molecules are linked through N3—H3···O15 hydrogen bonds into infinite zigzag chains extending along the *b* axis (Table 1, Fig. 2). In chains, molecules are regularly arranged in head-to-tail sequence allowing N—O···π stacking interactions which reinforce the chain cohesion (Fig. 2 and 3). These stacking forces are characterized by two N13—O15···C_g_^i, iv^ [symmetry code: i: -*x* + 1, *y* + 1/2, -*z* + 1; iv: -*x* + 1, *y* - 1/2, -*z* + 1] interactions with distances of 3.478 (1) Å and 3.238 (1) Å between the O15 atom and centroid, C_g_, of the (C7—C12) aromatic rings. Adjacent chains build the three dimensional network *via* C6—H6···O16 and C11—H11···Cl17 interactions (Table 1).

The title coumpound exhibits structural similarities with related structures (Bach *et al.*, 2001; Tsui *et al.*, 2013).

### Experimental

The title compound was obtained as a by-product from the reaction of 4-[hydroxy(4-nitrophenyl)methyl]-1,3-oxazolidin-2-one and benzenesulfonyl chloride with pyridine in chloroform. The synthesis process is described by Madesclaire *et al.* (2013). After isolation and purification by column chromatography, crystals suitable for X-ray analysis were obtained by slow evaporation from a mixture of ethyl acetate-cyclohexane (1:1 vol.) solution.

### Refinement

H atoms were all found in a difference map, but those bonded to C were refined using a riding model with *U*_iso_(H) = 1.2 *U*_eq_(C) and C—H = 0.93–0.98 Å. The H atom bonded to N was freely refined. The highest peak and the deepest hole in the difference Fourier map are located 0.70 and 1.02 Å, respectively from C11 and C8 atoms. The absolute structure was determined on the basis of 2348 Friedel pairs.

### Crystal data

**Table:**

C_10_H_9_ClN_2_O_4_	*F*(000) = 264
*M**_r_* = 256.64	*D*_x_ = 1.573 Mg m^−^^3^
Monoclinic, *P*2_1_	Melting point: 414 K
Hall symbol: P 2yb	Mo *K*α radiation, λ = 0.71073 Å
*a* = 7.2372 (1) Å	Cell parameters from 6817 reflections
*b* = 6.6726 (1) Å	θ = 4.0–39.2°
*c* = 11.7126 (2) Å	µ = 0.36 mm^−^^1^
β = 106.715 (1)°	*T* = 296 K
*V* = 541.71 (1) Å^3^	Block prism, colourless
*Z* = 2	0.52 × 0.49 × 0.34 mm

### Data collection

**Table:**

Bruker APEXII CCD diffractometer	6114 independent reflections
Radiation source: fine-focus sealed tube	5384 reflections with *I* > 2σ(*I*)
Graphite monochromator	*R*_int_ = 0.014
φ and ω scans	θ_max_ = 41.1°, θ_min_ = 4.2°
Absorption correction: multi-scan (*SADABS*; Bruker, 2012)	*h* = −11→13
*T*_min_ = 0.915, *T*_max_ = 1.000	*k* = −12→10
12895 measured reflections	*l* = −21→21

### Refinement

**Table:**

Refinement on *F*^2^	Secondary atom site location: difference Fourier map
Least-squares matrix: full	Hydrogen site location: inferred from neighbouring sites
*R*[*F*^2^ > 2σ(*F*^2^)] = 0.037	H atoms treated by a mixture of independent and constrained refinement
*wR*(*F*^2^) = 0.098	*w* = 1/[σ^2^(*F*_o_^2^) + (0.0548*P*)^2^ + 0.0254*P*] where *P* = (*F*_o_^2^ + 2*F*_c_^2^)/3
*S* = 1.06	(Δ/σ)_max_ < 0.001
6114 reflections	Δρ_max_ = 0.33 e Å^−^^3^
158 parameters	Δρ_min_ = −0.44 e Å^−^^3^
1 restraint	Absolute structure: Flack (1983), 2348 Friedel pairs
Primary atom site location: structure-invariant direct methods	Flack parameter: −0.03 (3)

### Special details

**Table:**

Geometry. All e.s.d.'s (except the e.s.d. in the dihedral angle between two l.s. planes) are estimated using the full covariance matrix. The cell e.s.d.'s are taken into account individually in the estimation of e.s.d.'s in distances, angles and torsion angles; correlations between e.s.d.'s in cell parameters are only used when they are defined by crystal symmetry. An approximate (isotropic) treatment of cell e.s.d.'s is used for estimating e.s.d.'s involving l.s. planes.
Refinement. Refinement of *F*^2^ against ALL reflections. The weighted *R*-factor *wR* and goodness of fit *S* are based on *F*^2^, conventional *R*-factors *R* are based on *F*, with *F* set to zero for negative *F*^2^. The threshold expression of *F*^2^ > σ(*F*^2^) is used only for calculating *R*-factors(gt) *etc*. and is not relevant to the choice of reflections for refinement. *R*-factors based on *F*^2^ are statistically about twice as large as those based on *F*, and *R*- factors based on ALL data will be even larger.

### Fractional atomic coordinates and isotropic or equivalent isotropic displacement parameters (Å^2^)

**Table:**

	*x*	*y*	*z*	*U*_iso_*/*U*_eq_	
O1	−0.27342 (14)	1.09892 (14)	0.00860 (7)	0.04046 (18)	
C2	−0.09939 (17)	1.16003 (16)	0.07729 (9)	0.03409 (18)	
N3	−0.04803 (15)	1.04959 (14)	0.17795 (9)	0.0379 (2)	
H3	0.035 (3)	1.073 (4)	0.234 (2)	0.064 (7)*	
C4	−0.18727 (13)	0.89934 (14)	0.18369 (8)	0.02872 (15)	
H4	−0.2438	0.9293	0.2484	0.034*	
C5	−0.33823 (17)	0.9297 (2)	0.06159 (11)	0.0423 (2)	
H5A	−0.3460	0.8116	0.0121	0.051*	
H5B	−0.4646	0.9553	0.0716	0.051*	
C6	−0.09944 (12)	0.68917 (13)	0.19899 (7)	0.02377 (12)	
H6	−0.0574	0.6564	0.1288	0.029*	
C7	0.07040 (11)	0.67131 (13)	0.30890 (7)	0.02336 (12)	
C8	0.04894 (13)	0.68896 (17)	0.42314 (7)	0.02948 (16)	
H8	−0.0736	0.7054	0.4323	0.035*	
C9	0.20770 (14)	0.68227 (17)	0.52297 (7)	0.03020 (16)	
H9	0.1936	0.6931	0.5992	0.036*	
C10	0.38832 (12)	0.65896 (14)	0.50598 (7)	0.02706 (14)	
C11	0.41443 (13)	0.64207 (18)	0.39412 (9)	0.03193 (17)	
H11	0.5374	0.6270	0.3855	0.038*	
C12	0.25405 (13)	0.64796 (18)	0.29494 (8)	0.02991 (16)	
H12	0.2691	0.6363	0.2190	0.036*	
N13	0.55799 (13)	0.64956 (14)	0.61094 (8)	0.03406 (16)	
O14	0.53442 (16)	0.6452 (3)	0.70939 (8)	0.0556 (3)	
O15	0.71821 (13)	0.6472 (2)	0.59423 (9)	0.0488 (2)	
O16	−0.0129 (2)	1.29556 (17)	0.04888 (10)	0.0528 (3)	
Cl17	−0.28820 (3)	0.51760 (4)	0.20713 (2)	0.03492 (6)	

### Atomic displacement parameters (Å^2^)

**Table:**

	*U*^11^	*U*^22^	*U*^33^	*U*^12^	*U*^13^	*U*^23^
O1	0.0460 (4)	0.0391 (4)	0.0291 (3)	0.0070 (3)	−0.0005 (3)	0.0078 (3)
C2	0.0433 (5)	0.0272 (4)	0.0324 (4)	0.0033 (4)	0.0119 (4)	0.0008 (3)
N3	0.0380 (4)	0.0299 (4)	0.0352 (4)	−0.0064 (3)	−0.0062 (3)	0.0051 (3)
C4	0.0271 (4)	0.0286 (3)	0.0258 (3)	0.0025 (3)	0.0002 (3)	0.0024 (3)
C5	0.0337 (5)	0.0426 (5)	0.0383 (5)	−0.0012 (4)	−0.0092 (4)	0.0089 (4)
C6	0.0232 (3)	0.0272 (3)	0.0201 (3)	0.0001 (2)	0.0049 (2)	0.0002 (2)
C7	0.0223 (3)	0.0262 (3)	0.0209 (3)	0.0023 (2)	0.0051 (2)	0.0020 (2)
C8	0.0229 (3)	0.0433 (5)	0.0221 (3)	0.0036 (3)	0.0063 (2)	0.0017 (3)
C9	0.0285 (4)	0.0399 (4)	0.0210 (3)	0.0030 (3)	0.0052 (3)	0.0024 (3)
C10	0.0248 (3)	0.0277 (3)	0.0250 (3)	0.0037 (3)	0.0013 (2)	0.0025 (3)
C11	0.0225 (3)	0.0415 (5)	0.0311 (4)	0.0062 (3)	0.0067 (3)	0.0010 (3)
C12	0.0256 (3)	0.0409 (4)	0.0239 (3)	0.0057 (3)	0.0083 (2)	0.0008 (3)
N13	0.0301 (3)	0.0315 (3)	0.0330 (4)	0.0043 (3)	−0.0029 (3)	0.0024 (3)
O14	0.0484 (5)	0.0801 (8)	0.0293 (4)	0.0045 (5)	−0.0032 (3)	0.0063 (5)
O15	0.0270 (3)	0.0631 (6)	0.0483 (5)	0.0074 (4)	−0.0018 (3)	0.0020 (5)
O16	0.0723 (7)	0.0370 (4)	0.0576 (6)	−0.0051 (4)	0.0321 (5)	0.0057 (4)
Cl17	0.03212 (10)	0.03645 (11)	0.03473 (10)	−0.00833 (9)	0.00727 (7)	−0.00087 (8)

### Geometric parameters (Å, º)

**Table:**

O1—C2	1.3480 (15)	C7—C12	1.3938 (12)
O1—C5	1.4310 (16)	C7—C8	1.3956 (11)
C2—O16	1.2004 (15)	C8—C9	1.3844 (13)
C2—N3	1.3487 (14)	C8—H8	0.9300
N3—C4	1.4367 (14)	C9—C10	1.3863 (13)
N3—H3	0.77 (2)	C9—H9	0.9300
C4—C6	1.5288 (12)	C10—C11	1.3811 (13)
C4—C5	1.5431 (13)	C10—N13	1.4679 (11)
C4—H4	0.9800	C11—C12	1.3866 (13)
C5—H5A	0.9700	C11—H11	0.9300
C5—H5B	0.9700	C12—H12	0.9300
C6—C7	1.5073 (11)	N13—O14	1.2136 (14)
C6—Cl17	1.8058 (9)	N13—O15	1.2303 (13)
C6—H6	0.9800		
			
C2—O1—C5	110.26 (8)	C4—C6—H6	109.0
O16—C2—O1	122.33 (11)	Cl17—C6—H6	109.0
O16—C2—N3	128.27 (12)	C12—C7—C8	119.65 (7)
O1—C2—N3	109.38 (9)	C12—C7—C6	118.66 (7)
C2—N3—C4	113.67 (9)	C8—C7—C6	121.59 (7)
C2—N3—H3	126.1 (18)	C9—C8—C7	120.85 (8)
C4—N3—H3	119.1 (18)	C9—C8—H8	119.6
N3—C4—C6	111.84 (8)	C7—C8—H8	119.6
N3—C4—C5	100.62 (8)	C8—C9—C10	118.05 (8)
C6—C4—C5	112.87 (8)	C8—C9—H9	121.0
N3—C4—H4	110.4	C10—C9—H9	121.0
C6—C4—H4	110.4	C11—C10—C9	122.49 (8)
C5—C4—H4	110.4	C11—C10—N13	118.76 (8)
O1—C5—C4	105.81 (9)	C9—C10—N13	118.74 (8)
O1—C5—H5A	110.6	C10—C11—C12	118.85 (8)
C4—C5—H5A	110.6	C10—C11—H11	120.6
O1—C5—H5B	110.6	C12—C11—H11	120.6
C4—C5—H5B	110.6	C11—C12—C7	120.10 (8)
H5A—C5—H5B	108.7	C11—C12—H12	119.9
C7—C6—C4	112.46 (7)	C7—C12—H12	119.9
C7—C6—Cl17	110.38 (6)	O14—N13—O15	123.18 (10)
C4—C6—Cl17	107.00 (6)	O14—N13—C10	118.97 (9)
C7—C6—H6	109.0	O15—N13—C10	117.84 (9)
			
C5—O1—C2—O16	177.34 (12)	Cl17—C6—C7—C8	−53.58 (10)
C5—O1—C2—N3	−3.94 (14)	C12—C7—C8—C9	−0.42 (15)
O16—C2—N3—C4	179.45 (12)	C6—C7—C8—C9	−176.72 (9)
O1—C2—N3—C4	0.83 (14)	C7—C8—C9—C10	0.44 (15)
C2—N3—C4—C6	122.37 (10)	C8—C9—C10—C11	−0.12 (16)
C2—N3—C4—C5	2.29 (13)	C8—C9—C10—N13	−179.40 (9)
C2—O1—C5—C4	5.25 (13)	C9—C10—C11—C12	−0.22 (17)
N3—C4—C5—O1	−4.35 (12)	N13—C10—C11—C12	179.06 (10)
C6—C4—C5—O1	−123.69 (10)	C10—C11—C12—C7	0.23 (17)
N3—C4—C6—C7	57.76 (10)	C8—C7—C12—C11	0.08 (16)
C5—C4—C6—C7	170.37 (8)	C6—C7—C12—C11	176.48 (10)
N3—C4—C6—Cl17	179.12 (6)	C11—C10—N13—O14	−173.10 (13)
C5—C4—C6—Cl17	−68.26 (9)	C9—C10—N13—O14	6.21 (16)
C4—C6—C7—C12	−110.50 (10)	C11—C10—N13—O15	7.17 (15)
Cl17—C6—C7—C12	130.09 (8)	C9—C10—N13—O15	−173.52 (11)
C4—C6—C7—C8	65.84 (11)		

### Hydrogen-bond geometry (Å, º)

**Table:**

*D*—H···*A*	*D*—H	H···*A*	*D*···*A*	*D*—H···*A*
N3—H3···O15^i^	0.77 (2)	2.32 (2)	3.095 (1)	179 (2)
C6—H6···O16^ii^	0.98	2.46	3.309 (2)	145
C11—H11···Cl17^iii^	0.93	2.83	3.582 (1)	139

Symmetry codes: (i) −*x*+1, *y*+1/2, −*z*+1; (ii) −*x*, *y*−1/2, −*z*; (iii) *x*+1, *y*, *z*.
